# Therapy-Emergent Drug Resistance to Integrase Strand Transfer Inhibitors in HIV-1 Patients: A Subgroup Meta-Analysis of Clinical Trials

**DOI:** 10.1371/journal.pone.0160087

**Published:** 2016-08-17

**Authors:** Jiangzhou You, Hongren Wang, Xiaojun Huang, Zhen Qin, Zhaomin Deng, Jun Luo, Baoning Wang, Mingyuan Li

**Affiliations:** Department of Microbiology, West China School of Preclinical and Forensic Medicine, Sichuan University, Chengdu, Sichuan, China; Azienda Ospedaliera Universitaria di Perugia, ITALY

## Abstract

**Background:**

Integrase strand transfer inhibitors (INSTIs) are a novel class of anti-HIV agents that show high activity in inhibiting HIV-1 replication. Currently, licensed INSTIs include raltegravir (RAL), elvitegravir (EVG) and dolutegravir (DTG); these drugs have played a critical role in AIDS therapy, serving as additional weapons in the arsenal for treating patients infected with HIV-1. To date, long-term data regarding clinical experience with INSTI use and the emergence of resistance remain scarce. However, the literature is likely now sufficiently comprehensive to warrant a meta-analysis of resistance to INSTIs.

**Methods:**

Our team implemented a manuscript retrieval protocol using Medical Subject Headings (MeSH) via the Web of Science, MEDLINE, EMBASE, and Cochrane Central Register of Controlled Trials databases. We screened the literature based on inclusion and exclusion criteria and then performed a quality analysis and evaluation using RevMan software, Stata software, and the Strengthening the Reporting of Observational Studies in Epidemiology (STROBE). We also performed a subgroup analysis. Finally, we calculated resistance rates and risk ratios (RRs) for the three types of drugs.

**Results:**

We identified 26 references via the database search. A meta-analysis of the RAL data revealed that the resistance rate was 3.9% (95% CI = 2.9%-4.9%) for the selected randomized controlled trials (RCTs). However, the RAL resistance rate reached 40.9% (95% CI = 8.8%-72.9%) for the selected observational studies (OBSs). The rates of resistance to RAL that were associated with HIV subtypes A, B, and C as well as with more complex subtypes were 0.1% (95% CI = -0.7%-0.9%), 2.5% (95% CI = 0.5%-4.5%), 4.6% (95% CI = 2.7%-6.6%) and 2.2% (95% CI = 0.7%-3.7%), respectively. The rates of resistance to EVG and DTG were 1.2% (95% CI = 0.2%-2.2%) and 0.1% (95% CI = -0.2%-0.5%), respectively. Furthermore, we found that the RRs for antiviral resistance were 0.414 (95% CI = 0.210–0.816) between DTG and RAL and 0.499 (95% CI = 0.255–0.977) between EVG and RAL. When RAL was separately co-administered with nuclear nucleoside reverse transcriptase inhibitors (NRTIs) or protease inhibitors (PIs), the rates of resistance to RAL were 0.2% (95% CI = -0.1%-0.5%) and 0.2% (95% CI = -0.2%-0.6%), respectively. The ten major integrase mutations (including N155H, Y143C/R, Q148H/R, Y143Y/H, L74L/M, E92Q, E138E/A, Y143C, Q148Q and Y143S) can reduce the sensitivity of RAL and EVG. The resistance of DTG is mainly shown in 13 integrase mutations (including T97T/A, E138E/D, V151V/I, N155H, Q148, Y143C/H/R, T66A and E92Q).

**Conclusions:**

Our results reveal that the DTG resistance rate was lower than the RAL resistance rate in a head-to-head comparison. Moreover, we confirmed that the EVG resistance rate was lower than the RAL resistance rate. In addition, our results revealed that the resistance rate of RAL was lower than that of efavirenz. The rates of resistance to RAL, EVG and DTG were specifically 3.9%, 1.2% and 0.1%, respectively. Compared with other types of antiviral drugs, the rates of resistance to INSTIs are generally lower. Unfortunately, the EVG and DTG resistance rates could not be compared because of a lack of data.

## Introduction

Many human immunodeficiency virus (HIV) therapies aim to inhibit multiple targets in the viral replication cycle. The application of antiviral drugs is widespread and includes nuclear nucleoside reverse transcriptase inhibitors (NRTIs), non-nucleoside reverse transcriptase inhibitors (NNRTIs), protease inhibitors (PIs), and fusion inhibitors; however, all of these drug classes have met with high resistance rates [1–4]. The resistance to anti-HIV-1 drugs not only renders existing therapies ineffective but also could cause that new patients who did not experienced therapy resistanted to existing agents. Encouragingly, integrase strand transfer inhibitors (INSTIs), the newest class of anti-HIV agents, exhibit high activity ininhibiting HIV-1 strains resistant to PIs, NRTIs and NNRTIs [5]. In 2007, the US Food and Drug Administration (FDA) approved the first INSTI (raltegravir; RAL); subsequently, elvitegravir (EVG) and dolutegravir (DTG) passed clinical trials and were licensed in 2012 and August 2013, respectively [6,7]. INSTIs suppress viral integration by blocking integrase (IN), which is the active site in the HIV-1 strand transfer step [8]. In the presence of an INSTI, the host’s repair enzymes recircularize the pro-viral DNA, and the viral replication cycle is aborted [9,10]. Compared with traditional anti-HIV agents, INSTIs significantly reduce the rate of fall in viral load of drug-naive and -experienced patients infected with HIV-1 [8]. RAL is well tolerated and displays satisfactory activity against HIV-1 strains. EVG, the second approved INSTI, also produces a significant inhibitory effect against the HIV-1 strand transfer step, but it must be taken with food and requires the co-administration of pharmacokinetic boosting agents [11,12]. Furthermore, clinical demonstrations have shown that first-generation INSTIs have a low genetic barrier to resistance [5,13] and that a cross-resistance between RAL and EVG has developed. Therefore, the development of next-generation INSTIs that show high activity against RAL- and EVG-resistant HIV-1 strains is critical [13]. DTG, a second-generation INSTI, was recently approved. Reports of DTG resistance are rare, and DTG is well tolerated by patients with HIV-1 who experience grade I adverse events [14]. In addition, DTG shows high efficacy in both naive and multi-experienced patients. Thus, DTG represents a more attractive therapeutic choice for patients infected with HIV-1. Although the guidelines for the use of antiretroviral agents in HIV-infected adults and adolescents have recommended INSTIs due to their treatment efficacy [15], a better understanding of INSTI resistance is critical for AIDS therapy. By analyzing the rates of resistance to INSTIs, this article aims to provide a reference for the continued clinical administration of AIDS therapies.

## Methods

### Database searches

This meta-analysis sought to include all of the literature regarding resistance to INSTIs published from January 2007 to March 2015. Our team implemented a manuscript retrieval protocol via Medical Subject Headings (MeSH), including the terms “integrase strand transfer inhibitors”, “integrase inhibitor”, “raltegravir”, “elvitegravir”, “dolutegravir”, “safety and efficacy” and “resistance” using the Web of Science, MEDLINE, EMBASE, Cochrane Central Register of Controlled Trials, Chinese Biomedical Literature, VIP and China National Knowledge Infrastructure (CNKI) databases. Furthermore, we manually searched the grey literature for the same period including abstracts from the fifth national conference on AIDS and viral hepatitis C held by the Chinese Medical Association and the seventh National Clinical Microbiology Academic Annual Meeting.

### Literature inclusion and exclusion criteria

#### Study types

We included randomized controlled trials (RCTs), non-RCT clinical trials, case-control studies, cohort studies, and case reports (n > 10). We excluded letters and pointer studies.

#### Literature types

We included clinical trials and pharmacological experiments encompassing clinical trials, and we excluded animal experiments, basic research experiments, basic pharmacological experiments, and experiments performed *in vitro*.

#### Patient types

Both outpatients and inpatients were included. Both English- and Chinese-language papers were included. The species of patients were not limited. The minimum patient age was 18 years old.

#### Intervention type

All of the patients included in this meta-analysis had been treated with INSTIs.

#### Outcome type

INSTI resistance was defined as genotypic or phenotypic resistance to INSTIs emerging during therapy, as indicated by protocol-defined virologic failure based on detection of the viral IN coding sequence or a positive resistance test [16]. A genotypic sensitivity score (GSS) of 0, a phenotypic sensitivity score (PSS) of 0, or both was also defined as indicating INSTI resistance emerging during therapy. The GSS and PSS, calculated separately in the resistance test, denote the numbers of drugs that showed genotypic sensitivity in patients infected with HIV as well as the number of drugs that showed phenotypic sensitivity among the patients [6].

### Inclusion and exclusion processes

First, two reviewers independently assessed the reliability of the title and the abstract according to the inclusion and exclusion criteria. Second, they evaluated the reliability of the literature that had passed the first screening step by assessingthe full text based on the inclusion criteria. We dealt with controversial literature by acquiring a third party expert’s opinion.

### Evaluating the quality of the methodology

For RCTs, we assessed the quality of the literature using the Cochrane Collaboration evaluation tool [17,18], which possesses 4 dimensions. These studies were scored using the evaluation tools, with each dimension representing one point. According to our scale, three or four points represented high quality, and scores of less than three points represented low quality. For observational studies (OBSs), we used the Strengthening the Reporting of Observational Studies in Epidemiology (STROBE) [19] criteria to evaluate quality. In particular, STROBE provides general reporting recommendations to describe OBSs and can therefore be used as a quality assessment tool for all OBSs (i.e., cohort studies, case-control studies and cross-sectional studies), and it STROBE has 22 dimensions. We scored the OBS literature according to STROBE, with each dimension representing one point. Scores of greater than 18 points represented high-quality, low-risk work, whereas scores that ranged from 15 to 18 represented medium-quality work, and scores of less than 15 represented low-quality work.

### Statistical analyses of the methodologies

We calculated antiviral resistance rates and their 95% CIs. The calculation of 95% CIs was based on the drug resistance rates, which obeyed the binomial distribution principle. For resistance rates of 0%, we calculated the 95% CIs by using the “rule of threes” principle [20]. The “rule of threes” principle deems that the upper bound 95% CI = 3/n (where n denotes the sample size). Heterogeneity was evaluated using the *I*^*2*^ test. If the *I*^*2*^ value was lower than 50%, then the data synthesis was completed using a fixed-effects model;otherwise, a random-effects model was used to calculate the resistance rates. When the P-value was lower than 0.05, the difference was considered significant. The arcsine method was used to produce funnel graphs [21]. During our analysis, we found that RAL and DTG were well suited to a head-to-head no-inferior test; thus, we compared the risk ratio (RR) of the two agents.

## Results

**Fig 1:**
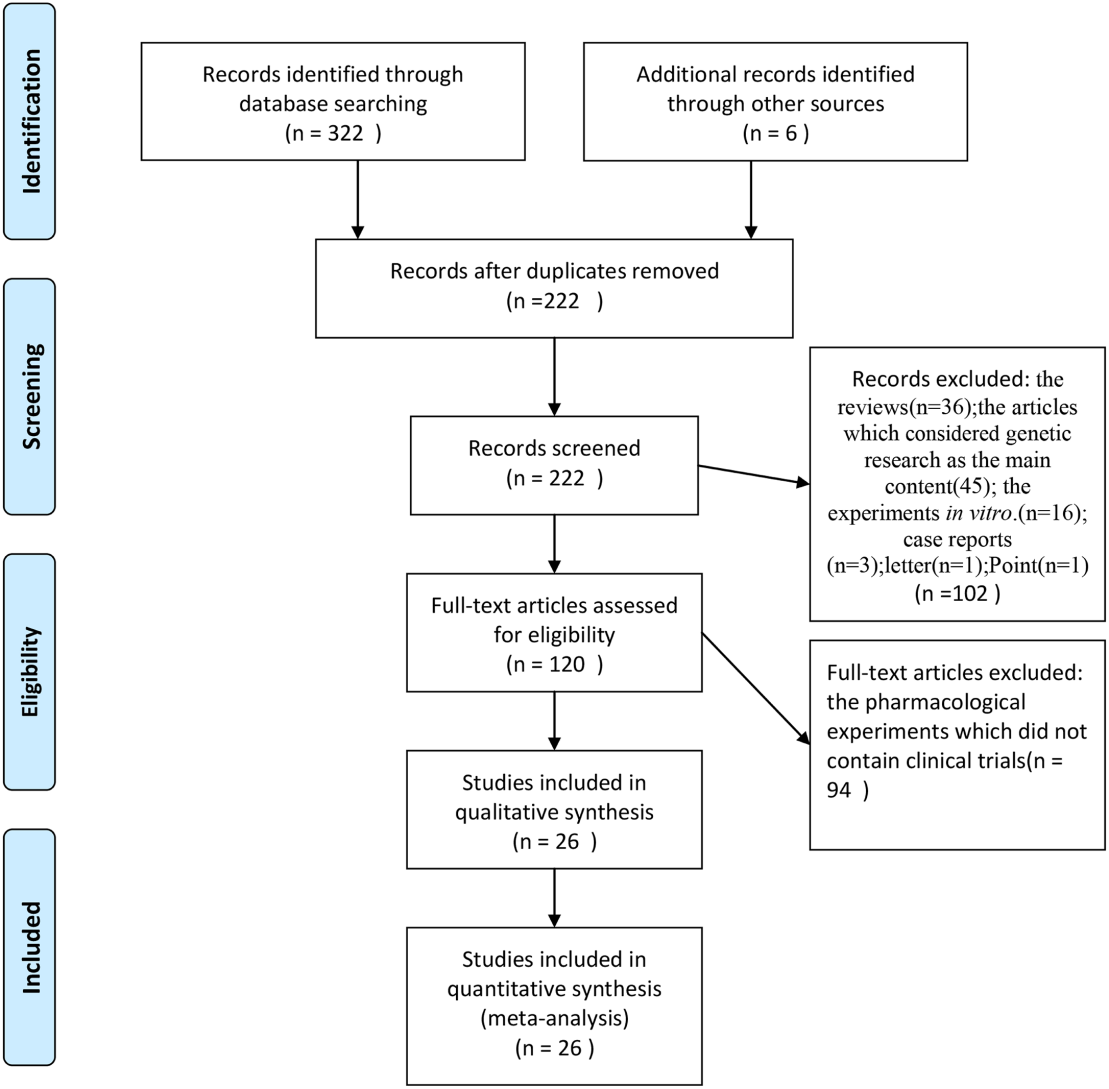
PRISMA diagram of the literature search. The PRISMA diagram illustrates the process through which literature was filtered, according to the designated inclusion and exclusion criteria. At each step, the reason for exclusion is indicated, where “n” represents the number of papers.

We identified 328 references via the database search and narrowed down the list to 26 papers that were deemed as reliable. The PRISMA diagram is presented in Fig 1.

**Fig 2:**
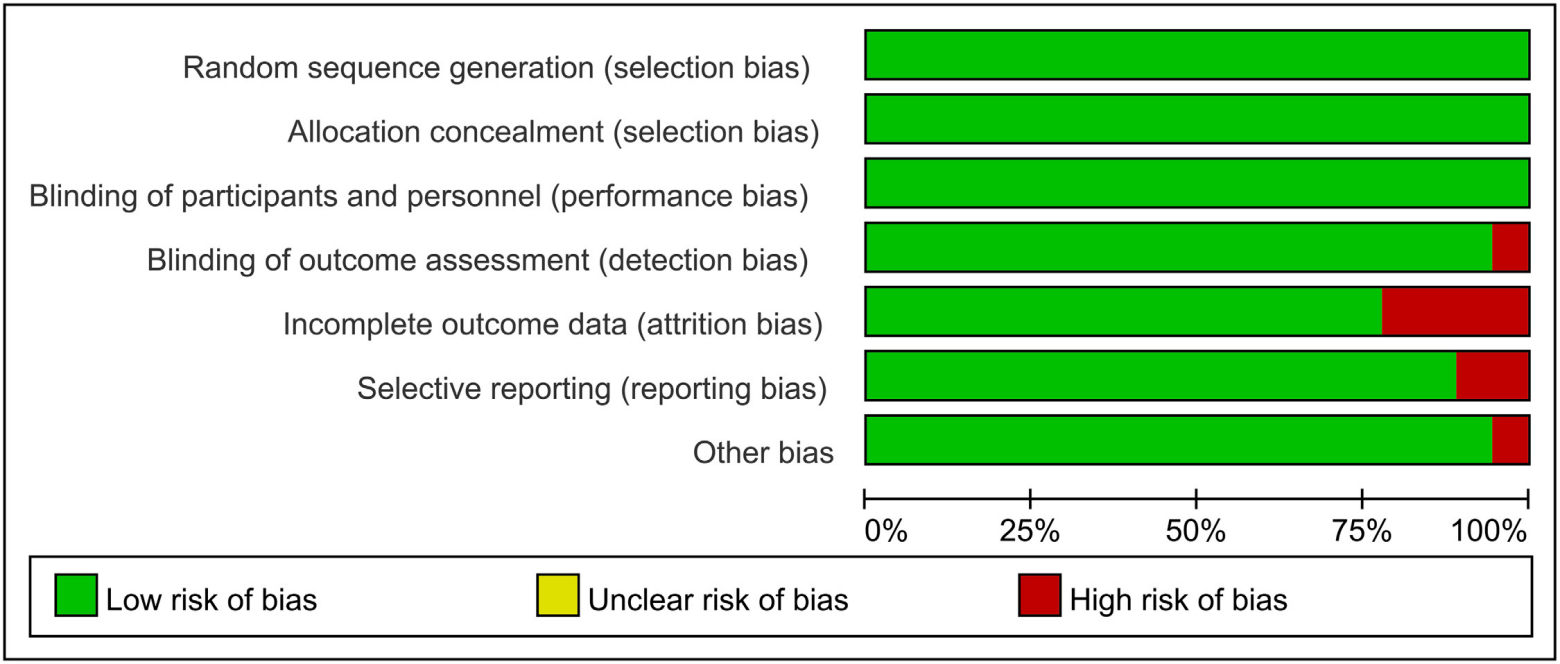
Quality evaluation of RCTs using RevMan software. In each dimension, the area of different colors represents the proportion of different publication biases derived from the included literatures. When no clear answer could be obtained for a dimension, it was classified as presenting a high risk of bias.

**Fig 3:**
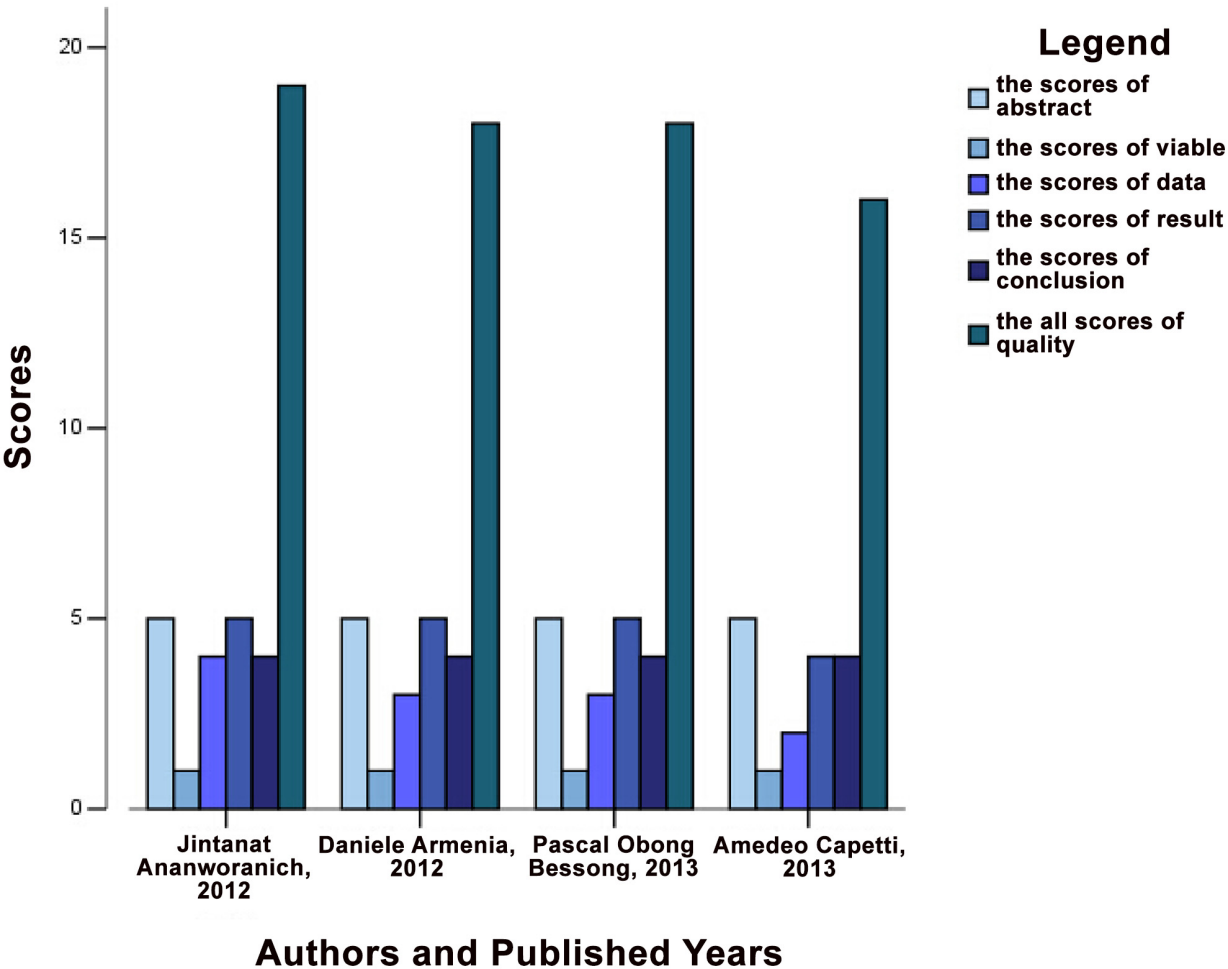
Histogram of quality evaluation for observational studies using STROBE. Different colors represent different dimensions in STROBE. The different regions on the x-axis illustrate different authors and publication dates. Each dimension was independently scored. “Sources” indicates the total points obtained for the quality evaluation process.

**Fig 4:**
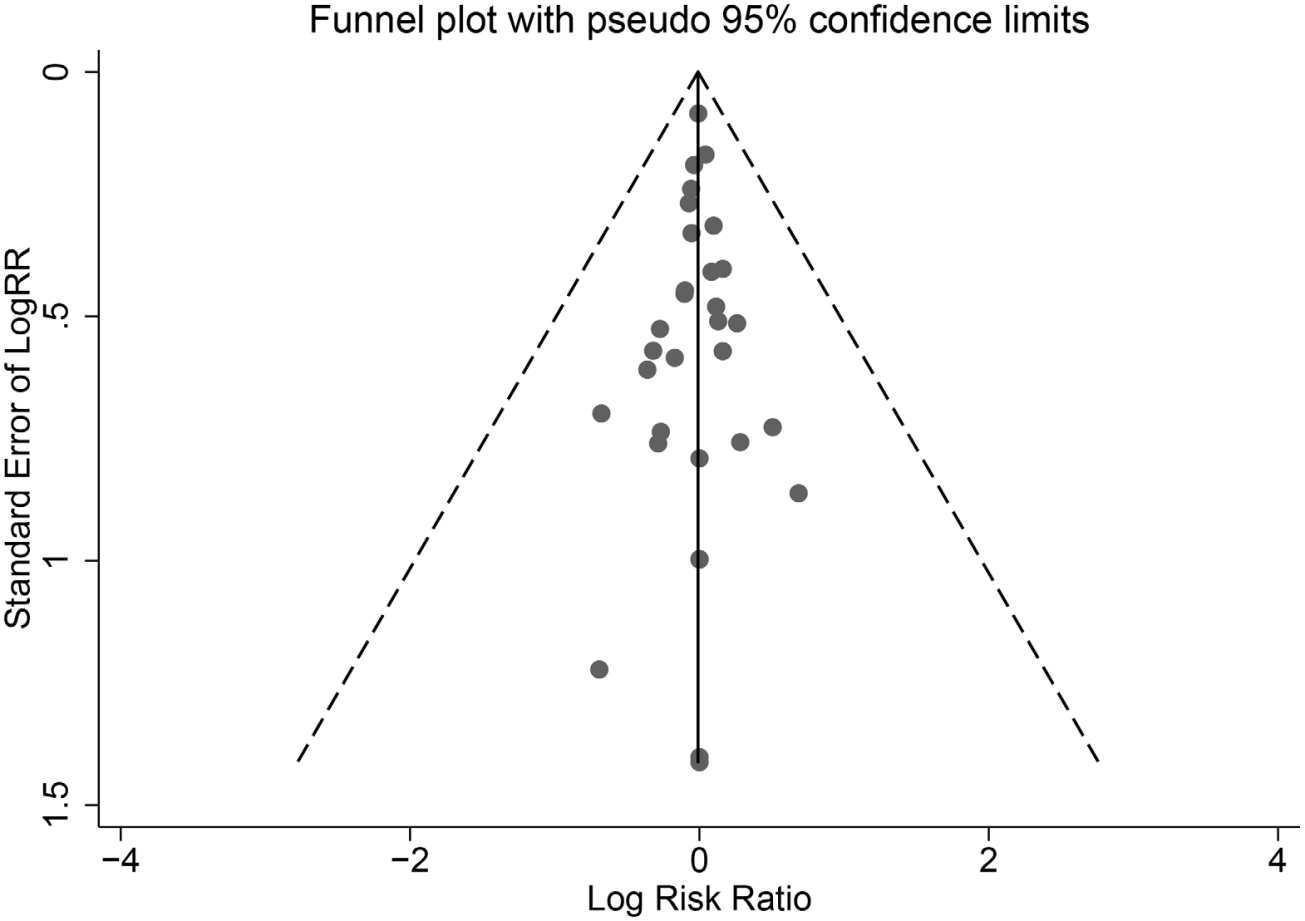
Funnel plot of the publication bias associated with the OBSs, as generated using Stata.

A total of 26 articles were selected, representing a cohort of 7,863 patients (Table 1). These articles coverd 21 double blind RCTs [5,6,11,22–34] and four OBSs [35–38]. Approximately 90% of the included literature was published from January 2010 to March 2015. In these studies, 17 articles reported only RAL resistance [6,11,22–28,35–38], whereas seven articles reported only EVG resistance [5,26,31–33]. Moreover, five articles reported only DTG resistance [11,15,22,34,39]. Three studies reported patients who were resistant to both RAL and EVG. Five studies performed a head-to-head non-inferiority test between RAL and DTG [11,15,22,34,39]. In terms of literature quality, all RCTs and three OBSs were of high quality, whereas one OBS was deemed to be of medium quality (Figs 2 and 3). The funnel plot was based on the literature describing RAL resistance. Fig 4 shows that the funnel plot is symmetrical, indicating that the publication bias of the literature was small. Because of the low number of quality studies describing DTG resistance and EVG resistance, a funnel plot cannot be constructed.

**Table 1:** Characteristics of the studies included in the systematic analysis.

Author (Year)	SD	TR	NPIP	IT	QES	PEG	PCG
**RAL group**							
**Lennox JL, 2009**	RCT	RAL	282	96 weeks	High	282	284
**Jeffrey L Lennox, 2010**	RCT	RAL	281	96 weeks	High	281	282
**Pedro Cahn, 2013**	RCT	RAL	362	48 weeks	High	361	354
**Francois Raffi, 2014**	RCT	RAL	77	96 weeks	High	401	404
**Francois Raffi, 2013 (1)**	RCT	RAL	411	96 weeks	High	411	411
**Joseph J Eron, 2013**	RCT	RAL	462	240 weeks	High	462	237
**Joseph J Eron Jr, 2011(1)**	RCT	RAL	382	48 weeks	High	386	389
**Joseph J Eron Jr, 2011(2)**	RCT	RAL	388	48 weeks	High	389	386
**Joseph J Eron, 2010 (1)**	RCT	RAL,	174	24 weeks	High	177	175
**Joseph J Eron, 2010 (2)**	RCT	RAL	176	24 weeks	High	176	178
**Jean-Michel Molina, 2012**	RCT	RAL	351	96 weeks	High	361	363
**Beatriz Grinsztejn, 2007 (1)**	RCT	RAL	44	24 weeks	High	44	45
**Beatriz Grinsztejn, 2007 (2)**	RCT	RAL	45	24 weeks	High	45	45
**Beatriz Grinsztejn, 2007 (3)**	RCT	RAL	45	24 weeks	High	45	45
**Jurgen K. Rockstroh, 2011 (1)**	RCT	RAL	219	96 weeks	High	219	230
**Jurgen K. Rockstroh, 2011 (2)**	RCT	RAL	59	96 weeks	High	59	47
**Jurgen K. Rockstroh, 2011 (3)**	RCT	RAL	416	96 weeks	High	416	219
**Jurgen K. Rockstroh, 2011 (4)**	RCT	RAL	39	96 weeks	High	39	15
**Jurgen K. Rockstroh, 2013**	RCT	RAL	282	48 weeks	High	282	284
**Y. Yazdanpanah, 2009**	RCT	RAL	100	48 weeks	High	100	100
**Babafemi Taiwo, 2011**	RCT	RAL	112	48 weeks	High	112	112
**Roy T. Steigbigel, 2008 (1)**	RCT	RAL	232	48 weeks	High	232	118
**Roy T. Steigbigel, 2008 (2)**	RCT	RAL	230	48 weeks	High	230	119
**Joseph J Eron, 2013**	RCT	RAL	462	240 weeks	Medium	462	237
**Slim Fourati1, 2015**	RCT	RAL	306	48 weeks	High	306	306
**Richard Elion, MD, 2013**	RCT	RAL	351	48 weeks	High	351	351
**Amedeo Capetti, 2013**	OBS	RAL	258	206 weeks	Medium	258	
**Jintanat Ananworanich, 2012**	OBS	RAL	19	119 days	High	19	
**Daniele Armenia, 2012**	OBS	RAL	23	24 weeks	High	23	
**Pascal Obong Bessong, 2013**	OBS	RAL	127	24 weeks	High	127	
**Vero´nica Briz, 2011**	RT	RAL	19	80.1weeks	High	19	
**EVG group**							
**Calvin Cohen, 2011**	RCT	EVG	50	48 weeks	High	50	25
**Andrew R. Zolopa, 2010 (1)**	RCT	EVG	75	48 weeks	High	75	73
**Andrew R. Zolopa, 2010 (2)**	RCT	EVG	75	48 weeks	High	75	73
**Andrew R. Zolopa, 2010 (3)**	RCT	EVG	74	48 weeks	High	74	73
**Paul E Sax, 2012**	RCT	EVG	348	48 weeks	High	348	352
**Jean-Michel Molina, 2012**	RCT	EVG	361	96 weeks	High	361	351
**Jose R Arribas, 2014**	RCT	EVG	293	96 weeks	High	293	145
**Slim Fourati1, 2015**	RCT	EVG	306	48 weeks	High	306	306
**Richard Elion, MD, 2013**	RCT	EVG	351	48 weeks	High	351	351
**DTG group**							
**Antonella Castagna, 2014**	RCT	DTG	183	24 weeks	High	183	183
**Francois Raffi, 2013 (1)**	RCT	DTG	411	96 weeks	High	411	411
**Pedro Cahn, 2013**	RCT	DTG	357	48 weeks	High	361	354
**Francois Raffi, 2013 (2)**	RCT	DTG	411	96 weeks	High	411	411
**Joseph J. Eron, 2012 (1)**	RCT	DTG	27	24 weeks	High	27	24
**Joseph J. Eron, 2012 (2)**	RCT	DTG	24	24 weeks	High	24	27

SD, study design; TR, treatment regimen; NPIP, No. of patients in the ITT population; IT, intervention time; QES, quality evaluation of the study; PEG, patients in the experimental group; PCG, patients in the control group; RT, retrospective study. “n” indicates the number of trials in the same literature. “OBS” indicates an observational study. “[n, n%]” indicates the number of patients and their proportion.

**Fig 5:**
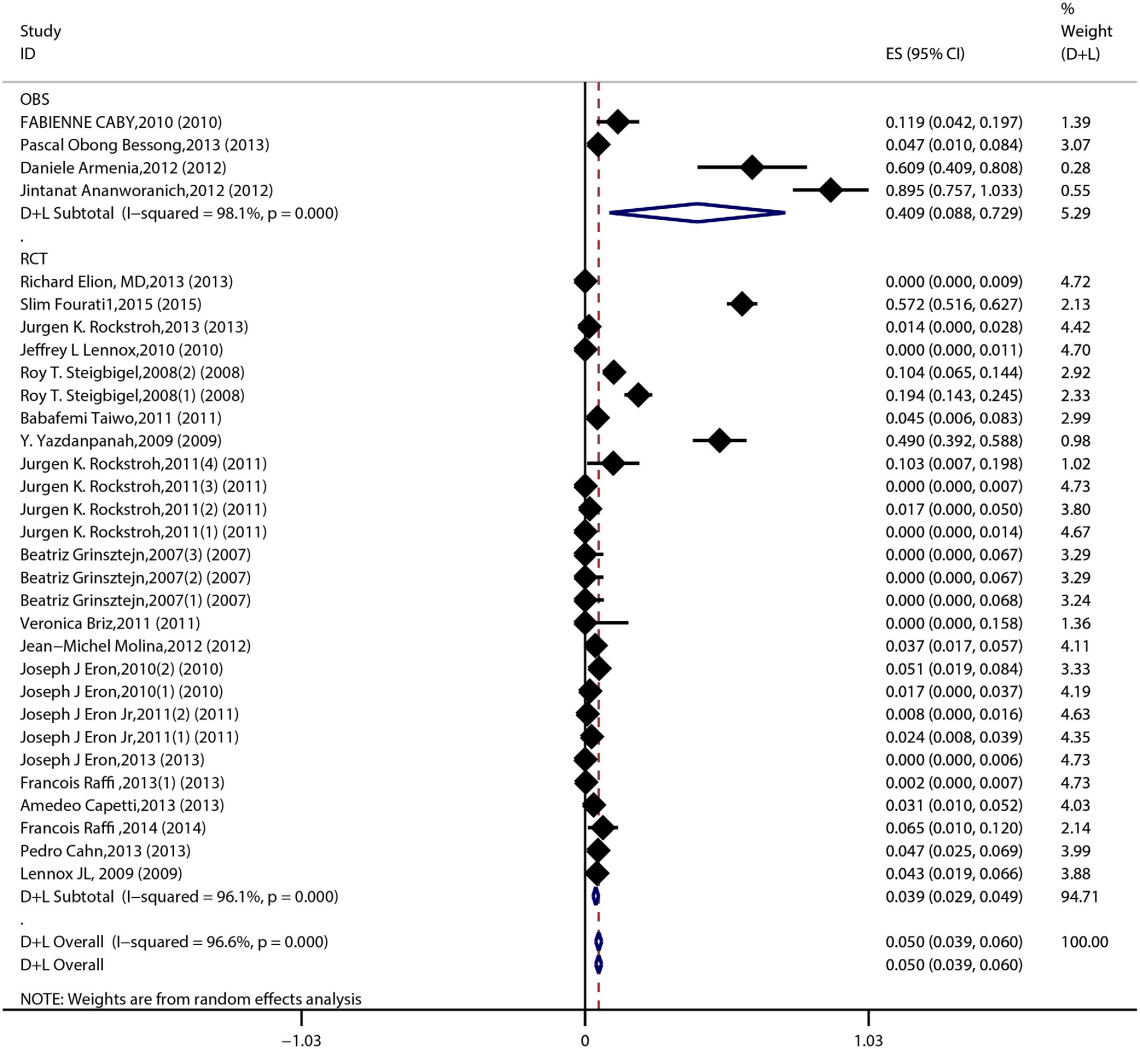
Forest plot for the rate of resistance to RAL, as determined using Stata. RCTs and OBSs formed the basis of this classification. “OBS” indicates the observational nature of the study. ES denotes the effect value (i.e., resistance rate). The important indicator *I*^*2*^ was used to evaluate the heterogeneity of the data. A hollow diamond represents the result of the meta-analysis. “n” indicates the different trial numbers for a given piece of literature. A black diamond represents the resistance rate for each trial. The width of the horizontal line passing through the black diamond denotes the 95% CI. The meta-analysis was completed using a random-effects model.

**Fig 6:**
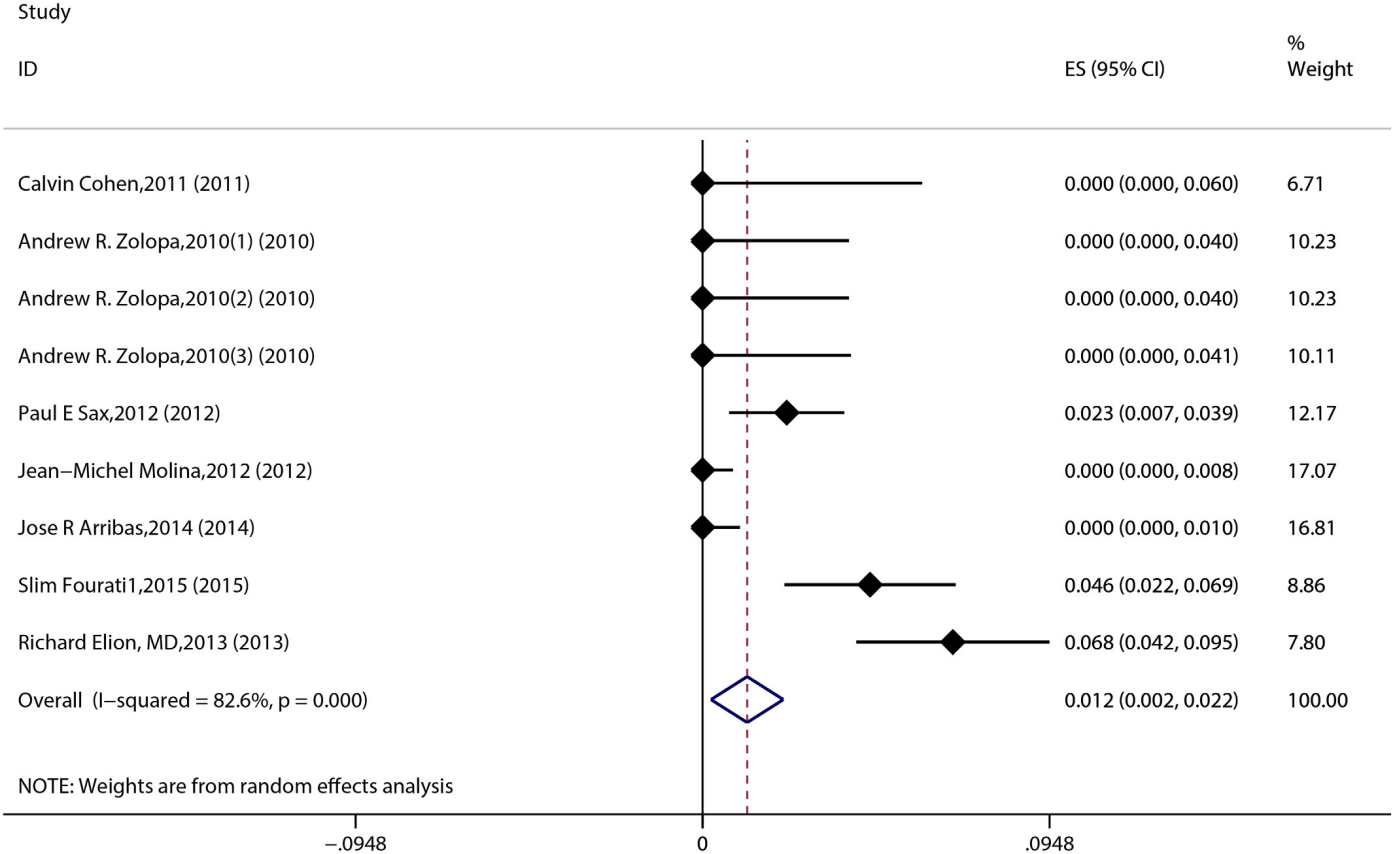
Forest plot for the rate of resistance to EVG, as determined using Stata.

**Fig 7:**
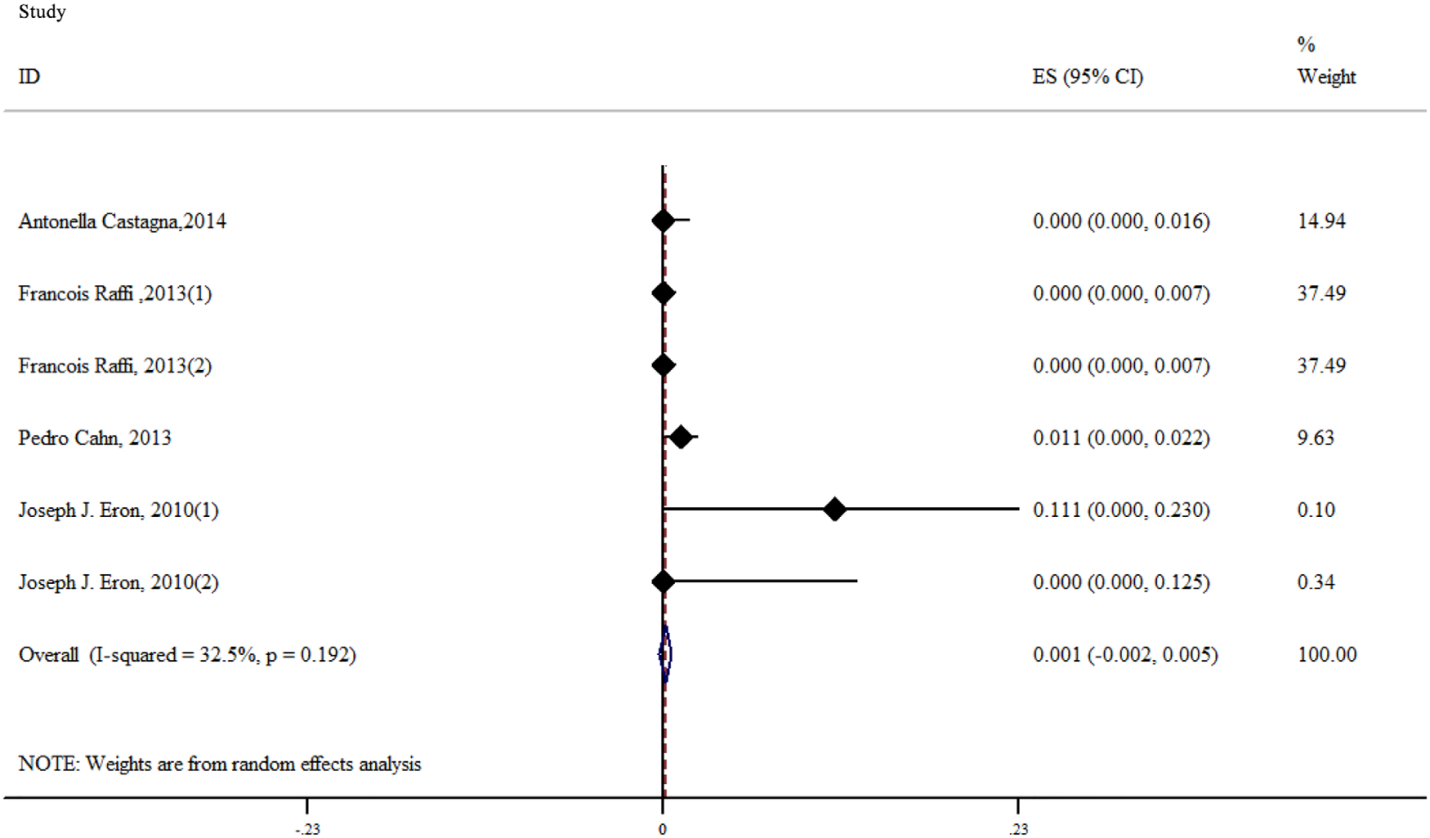
Forest plot for the rate of resistance to DTG, as determined using Stata.

**Fig 8:**
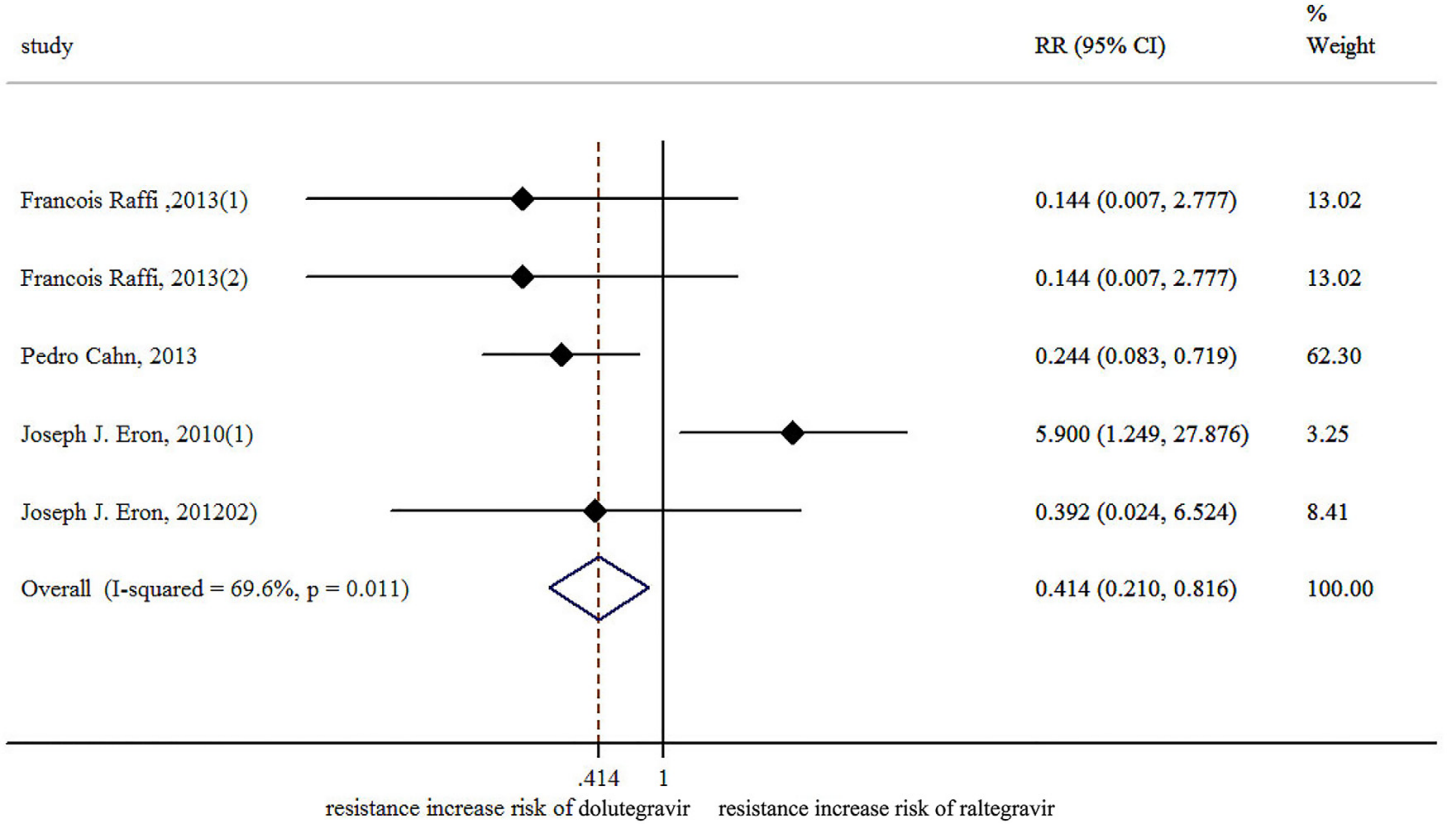
Forest plot for the RR between DTG and RAL, as determined using Stata. The RR was obtained using the following formula: DTG resistance rate divided by the RAL resistance rate. The data calculation was performed according the Mantel-Haenszel method.

**Fig 9:**
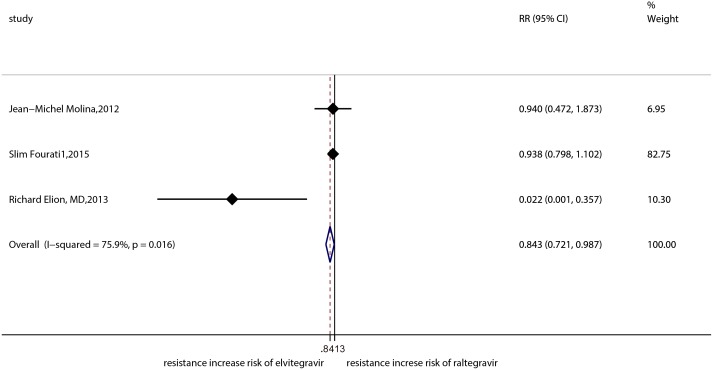
Forest plot for the RR between EVG and RAL, as determined using Stata.

**Fig 10:**
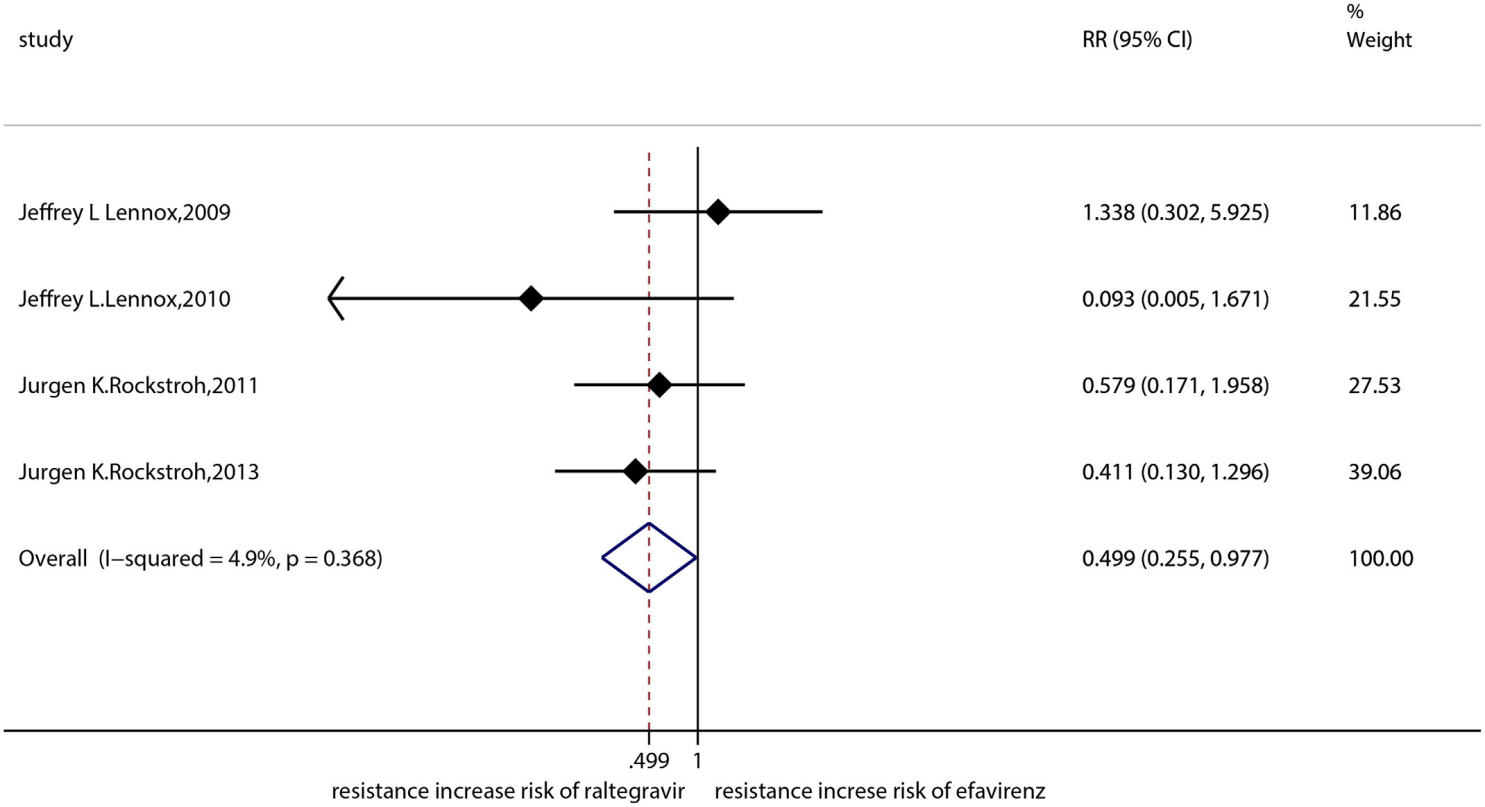
Forest plot for the RR between RAL and efavirenz, as determined using Stata.

The result of the meta-analysis for RAL indicated that the resistance rate was 3.9% (95% CI = 2.9%-4.9%) for the RCTs. However, the RAL resistance rate reached up to 40.9% (95% CI = 8.8%-72.9%) for the OBSs. Furthermore, the estimated heterogeneity (*I*^*2*^) of the two types of studies reached 96.1% and 98.1%, respectively (Fig 5). The meta-analysis results for the 9 RCTs in the EVG group showed that the resistance rate was 1.2% (95% CI = 0.2%-2.2%; Fig 6), the meta-analysis results for the 6 RCTs in the DTG group showed that the resistance rate was 0.1% (95% CI = -0.2%-0.5%; Fig 7). Next, we compared the resistance rates across the three types of agents prior to a head-to-head comparison of RAL and DTG; this test showed that the DTG resistance rate was 0.414 times the RAL resistance rate (RR = 0.414; 95% CI = 0.210–0.816; Fig 8). Thus, this result illustrated that the DTG resistance rate was lower than th RAL resistance rate. The RR for the resistance rate was 0.843 (95% CI = 0.721–0.987) when EVG was compared with RAL (Fig 9). This figure was 0.499 (95% CI = 0.255–0.977) when RAL was compared with efavirenz (Fig 10). The result of comparing RAL group with EVG group explained why the resistance rate observed for EVG was lower than that observed for RAL. At the same time, our result revealed that the RAL resistance rate was lower than the efavirenz resistance rate. However, the comparison of the EVG group with the DTG group was not supported by empirical evidence. Thus, the EVG and DTG resistance rates could not be compared. More evidence is needed to improve the accuracy of this test.

**Fig 11:**
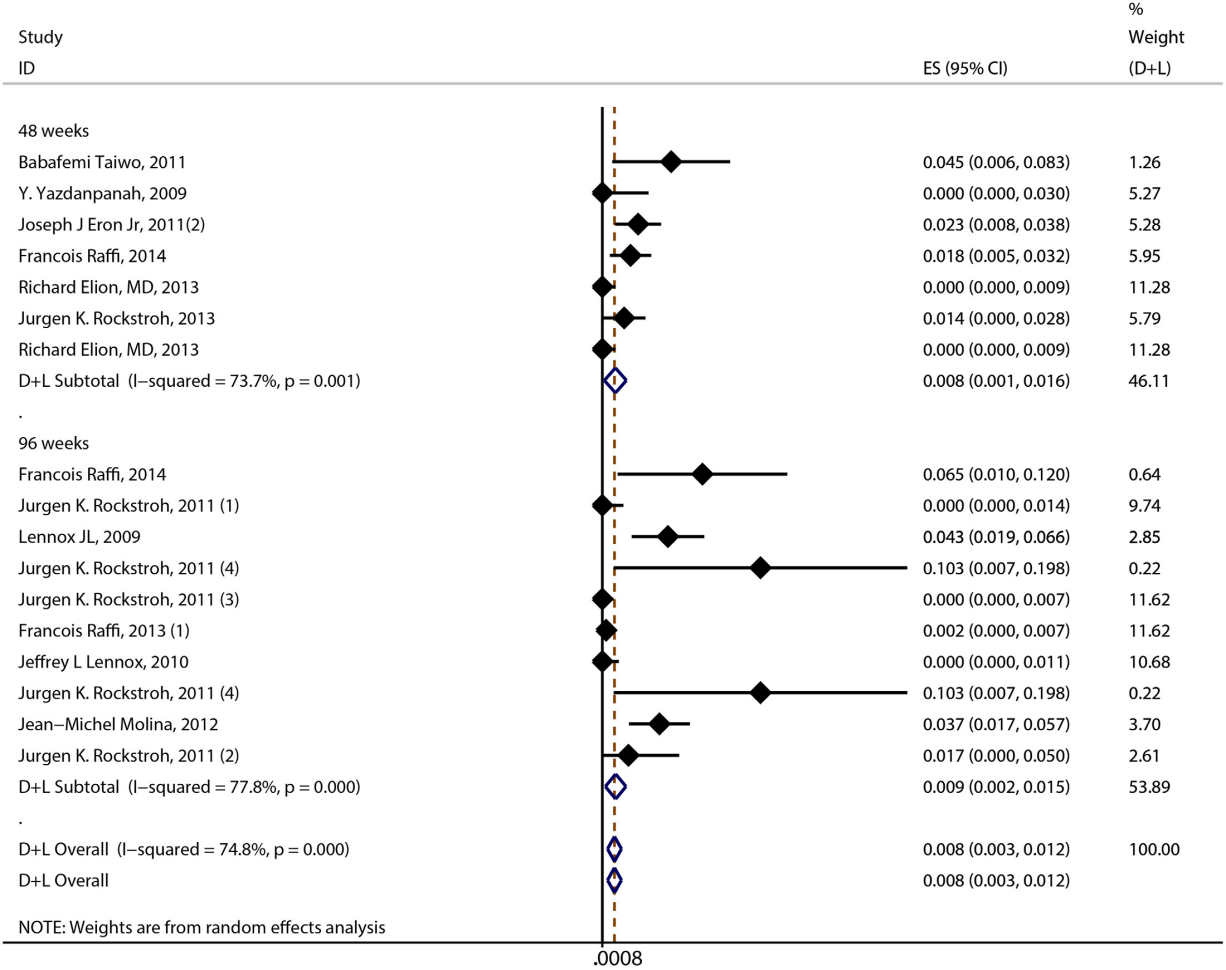
Forest plot for the rate of resistance to RAL, based on therapeutic time subgroup analysis.

Gven that administrated time of the antiviral agents for HIV has a significant impact on resistance rates, this parameter was usedas a basis of classification for subgroup analysis in this research. When RAL was administrated with AIDS patients, the subgroup analysis result showed that these resistance rates were 0.8%(95% CI = 0.1%-1.6%) and 0.9%(95% CI = 0.2%-1.5%) when patients were treated for 48 weeks and 96 weeks, respectively. The estimated heterogeneity (*I*^*2*^) of the two groups studies were 73.7% and 77.8%, respectively (Fig 11). In 240 weeks, the data had only one and it could not be mergered with others. In terms of EVG and DTG, the data which originated from resistance shown discrete distribution. So, we can't analyze their resistance rates.

**Fig 12:**
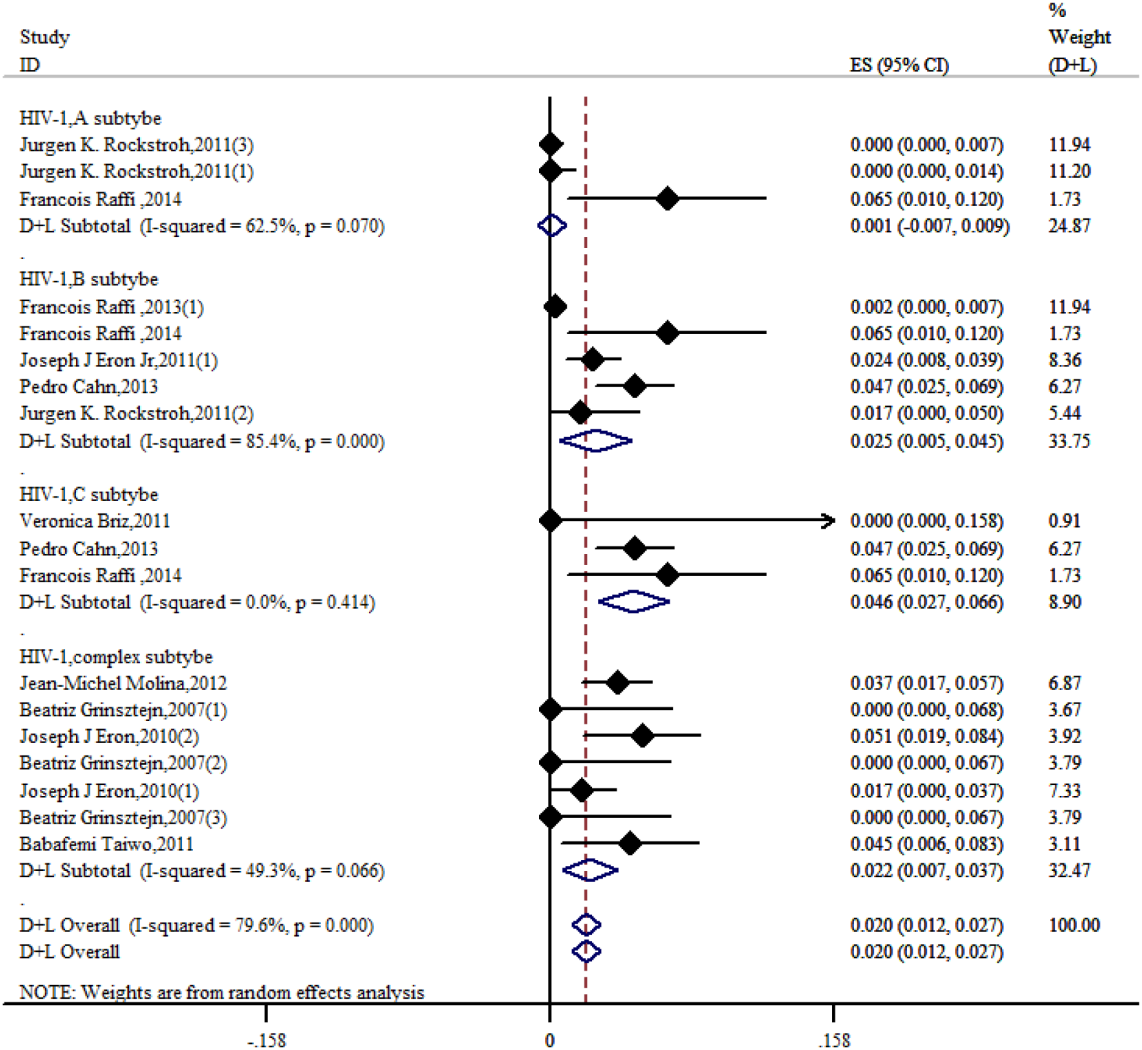
Forest plot for the rate of resistance to RAL, based on HIV-1 subtypes subgroup analysis. The complex subtype contains the all subtypes except for the A, B and C subtypes. When the same data contained different subtypes, it was assigned to different subtypes according to weight.

Because of the high heterogeneity of the RAL group, we conducted a subgroup analysis. The results of HIV-1 subtype testing for the A, B, and C subtypes as well as the complex subtype (contains D, E, F and G) showed that the corresponding resistance rates were 0.1% (95% CI = -0.7%-0.9%), 2.5% (95% CI = 0.5%-4.5%), 4.6% (95% CI = 2.7%-6.6%) and 2.2% (95% CI = 0.7%-3.7%), respectively (Fig 12). The estimated heterogeneity of each subgroup, with the exception of the B subtype, was small.

**Fig 13:**
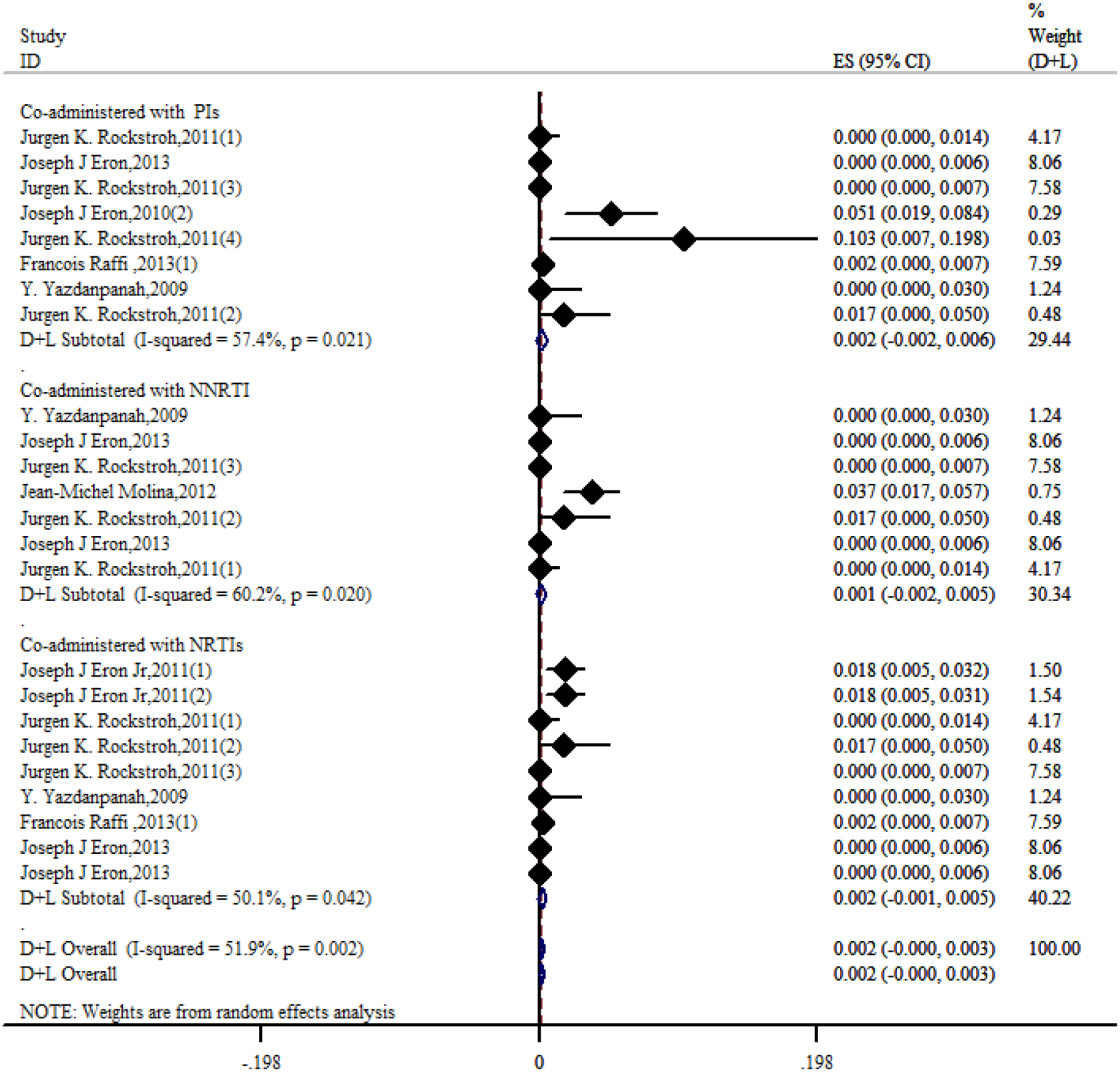
Forest plot for the rate of resistance to RAL based on cross-resistance subgroup analysis.

Under the currently considered therapeutic regimens, when RAL was co-administered with NNRTIs, PIs or NRTIs, we performed a subgroup analysis to determine the cross-resistance of INSTIs combined with an additional anti-HIV-1 drug. As shown in Fig 13, RAL had the lowest resistance rate when co-administered with an NNRTI, with value of 0.1% (95% CI = -0.2%-0.5%). Higher resistance rates were related to RAL when it was co-administered with either NRTIs or PIs, yielding rates of 0.2% (95% CI = -0.2%-0.6%) and 0.2% (95% CI = -0.1%-0.5%), respectively. We did not gather evidence regarding the cross-resistance for EVG and DTG when separately co-administered with other drugs.

## Discussion

INSTIs, which suppress the RNA replication of HIV-1 strains, appeared on the market in 2007 and have quickly become staples of the anti-HIV-1 drug arsenal. The clinical use of INSTIs represented a milestone that appeared 10 years after highly active antiretroviral therapy (HAART) was introduced to treat AIDS. For both naive and experienced patients, clinical trials have shown that INSTIs are safe and effective. For patients who cannot use other therapeutic agents, INSTIs inhibit viral reproduction and accelerate the reduction of plasma HIV-1 RNA levels. Moreover, INSTIs exhibit strong activity against multi-drug-resistant HIV-1 strains and have a synergistic effect when co-administered with other drug types. Thus, in the context of a disclosed therapeutic regimen, INSTIs have been prioritized as a first-line treatment for AIDS. Recently, it was reported that HIV-1 can become resistant to INSTIs during therapy. Moreover, reports of resistance genes were also rapidly emerging. The ten major integrase mutations(including N155H, Y143C/R, Q148H/R, Y143Y/H, L74L/M, E92Q, E138E/A, Y143C, Q148Q and Y143S) could reduce the sensitivity of RAL and EVG. Secondary integrase mutations(G140S/G and T97T/A) usually appeared together with Q148H/R and Y143Y/H. The resistance of DTG mainly shown in 13 integrase mutations(including T97T/A, E138E/D, V151V/I, N155H, Q148, Y143C/H/R, T66A and E92Q). After INSTIs which were represented by RAL applied to clinical treatment, consistented with reverse transcriptase inhibitors and protease inhibitors, they had inevitably emerged resistance. At present, however, a systematic summary of resistance rates for INSTIs has not been generated.

The primary focus of this meta-analysis was to determine the incidence of drug resistance among different RAL subgroups as well as the incidence of drug resistance for the EVG and DTG. Furthermore, we compared the rates of resistance to the three types of agents. Our results revealed that the DTG resistance rate was lower than the RAL resistance rate in a head-to-head comparison. We also confirmed that the EVG resistance rate was lower than the RAL resisitance rate. A prior study found the head-to-head no-inferiority test for directly comparing the rates of resistance to INSTIs was not used for the same studies in the literature [40]. This method worked well for controlling the baseline consistency of the two sides such that we were able to draw more convincing conclusions. Efavirenz is often the first and most widespread anti-HIV medication used in highly active antiretroviral therapies. Our analysis revealed that the RAL resistance rate was lower than the efavirenz resisitance rate; this conclusion might lead to clear guidelines for clinical doctors regarding the choice of an antiviral drug. The resistance rates of RAL, EVG and DTG were 3.9%, 1.2% and 0.1%, respectively. Five published papers have report that HIV-1 strains are group-resistant to RAL and DTG. This result sends a clear signal to clinicians: HIV-1 strains that are resistant to first- and second-generation INSTIs have emerged, and the clinical utility of INSTIs is on the decline. Compared with the other types of antiviral drugs, INSTIs generally display lower resistance rates; this conclusion is based on numerous RCTs. Moreover, the quantity of patients included in the present meta-analysis is unprecedented. Thus, the credibility of the conclusions drawn here is high. When HIV-1 is not optimally suppressed during treatment, resistance mutations accumulate that were dependent on subtype in the RAL group. The results of the HIV-1 subtype determination for the A, B, C, and complex (D, E, F and G) subtypes showed that their resistance rates were 0.1%, 2.5%, 4.6% and 2.2%, respectively. The effect of RAL on different subtypes is a critical issue for analysis because patients undergoing this treatment might be at risk of failing INSTI therapy. When RAL was separately co-administered with NNRTIs, NRTIs or PIs, the rates of resistance to RAL were 0.1%, 0.2%, and 0.2%, respectively, resulting in an extension of the treatment regimen. A conclusion regarding resistance rates could not be made because of a lack of data for EVG compared with DTG. This present meta-analysis encompasses all of the literature published to date; therefore, it is illustrative of current resistance trends. Although this article lacks funnel plots concerning EVG and DTG resistance, we have included literature concerning EVG and DTG in the light of their scheduled clinical design programs. Given this fact, the reliability of our conclusions is unaffected.

Several clinical trials have reported the emergence of phenotypes and genotypes associated with EVG resistance, but few studies have reported DTG resistance. Strict inclusion and exclusion criteria were applied for our literature review, which might have played a negative role in terms of reducing the quantity of the literature analyzed. Given the rates of resistance to INSTIs, these disadvantageous factors impeded our ability to draw certain conclusions. Thus, we will attempt to collect more evidence concerning EVG and DTG. Because articles implementing head-to-head non-inferiority tests were rare, we did not use this method to compare the resistance rates between EVG and DTG. Compared with OBSs, the data obtained via RCTs were more realistic. However, extrapolation from RCTs suffers from many restrictions and was therefore weaker than that from the OBSs. In addition, the literature types included were not balanced (i.e., we included more RCTs than OBSs). Thus, the proportion of OBSs must be improved to strengthen our conclusions. However, because formal clinical indicators are rarely used in OBSs, we excluded many such studies in the current analysis. Compared with RCTs, the quality of OBSs was lower for the RAL group; this disparity might explain why OBSs exhibited significant heterogeneity. At the same time, this difference might have revealed the incredible result that the resistance rate of the OBSs was significantly higher than that of the RCTs. Thus, an overvaluation of the resistance rate of the OBSs is possible. Regretfully, in this research, the quantity of timepoints was too little. So, we can only obtain the current conditions of the resistace rates which derived from each timepoints and had no scientific method to estimate popular trend of resistance which was affected by the therapeutic time.

Currently, published articles regarding INSTI resistance are primarily reviews of pharmacological studies [40–42] that do not address INSTI resistance rates in clinical trials. In addition, the experimental data extracted from these articles do not originate from large-scale or multi-center clinical trials. Thus, these articles contribute little to understanding of the clinical administration of INSTIs for AIDS therapy. Based on RCTs, we implemented a meta-analysis and successfully isolated the most accurate research on INSTI resistance. In terms of resistance rates, an apparent disparity was found between the article and other literatures reporting INSTIs resistance [43,44]. The leading cause was that the other literature mostly used data from small samples and primarily adopted OBSs, invariably suffering from uncontrollable interference. This view was clearly demonstrated in our analysis(i.e., the resistance rate of 40.9% for the OBSs). Of course, due to discrepant grouping methods, two types of researches selected heterogenetic objects and these studies lacked of selectivity, which were also the main causes which lead to the phenomenon. In addition, the method used to measure resistance in patients relied on the detection of gene mutations in other studies. Moreover, glaring errors were found in other studies (i.e., patients were deemed resistant to INSTIs when they did not respond to the medication, presented drug interactions or exhibited individual differences with regard to pharmacokinetic parameters). However, the current article adopted the GSS and PSS as the indicators used to evaluate the emergence of INSTI resistance in patients. Thus, the conclusions of this meta-analysis are more scientific, reliable and clinically significant.

## Supporting Information

S1 PRISMA Checklist PRIMSA Checklist: 2009. (DOC)
